# Associations of multiregional OCTA metrics and systemic hematologic biomarkers With 12-month diabetic retinopathy progression

**DOI:** 10.3389/fmed.2026.1951011

**Published:** 2026-10-02

**Authors:** Lina Ge, Shenghai Huang, Weihe Zhou, Lijin Huang, Chaoying Ye, Ying Huang, Xueyi Chen

**Affiliations:** 1 Department of Ophthalmology, The First Affiliated Hospital of Xinjiang Medical University, Urumqi, Xinjiang, China; 2 National Clinical Research Center for Ocular Diseases, Eye Hospital, Wenzhou Medical University, Wenzhou, China

**Keywords:** diabetic retinopathy, disease progression, hematologic biomarkers, optical coherence tomography angiography (OCTA), vessel density

## Abstract

**Background:**

This study investigated the associations of multiregional macular and peripapillary OCTA metrics and systemic hematologic biomarkers with 12-month diabetic retinopathy progression and evaluated whether their combination improved prognostic discrimination.

**Methods:**

This single-center longitudinal cohort study included 55 adults with type 2 diabetes and established DR, contributing 108 eyes. Baseline macular and peripapillary vessel density parameters were obtained by OCTA, and peripheral blood laboratory markers were measured. DR progression was defined as worsening by at least two steps on the Diabetic Retinopathy Severity Scale (DRSS) at 12 months. Generalized estimating equation (GEE) models were used to account for within-participant inter-eye correlation. Univariable and multivariable GEE analyses were conducted to identify baseline factors independently associated with DR progression. Receiver operating characteristic analysis was performed for the individual markers and combined models.

**Results:**

During follow-up, 52 eyes (48.15%) showed DR progression and 56 eyes (51.85%) did not. In the final multivariable GEE model, higher temporal peripapillary VD (adjusted OR, 0.88; 95%CI, 0.81–0.95), higher deep VD within the 1–3-mm parafoveal annulus (adjusted OR, 0.86; 95% CI, 0.78–0.96; *P* = 0.01), and higher lymphocyte count (adjusted OR, 0.31; 95% CI, 0.11–0.87; *P* = 0.03) were associated with lower odds of progression. The AUCs were 0.72 for temporal peripapillary VD, 0.70 for deep parafoveal VD, and 0.70 for lymphocyte count. The OCTA model combining the two VD parameters achieved an AUC of 0.80 (95% CI, 0.72–0.88), whereas the OCTA-plus-lymphocyte model achieved an AUC of 0.84 (95% CI, 0.77–0.92). After internal validation by participant-level cluster bootstrap (500 resamples), the optimism-corrected AUC of the OCTA-plus-lymphocyte model was 0.83 (apparent AUC 0.84; optimism ≤0.012), and the 10-fold participant-level cross-validated AUC was 0.81 for the combined model vs. 0.78 for the OCTA-only model.

**Conclusion:**

Lower temporal peripapillary VD, lower deep parafoveal VD, and lower peripheral blood lymphocyte count were associated with 12-month DR progression. Their combination demonstrated the highest apparent discrimination in this cohort. As this is a single-center exploratory cohort without external validation, these discriminative estimates should be regarded as apparent, and the model requires confirmation in larger, multicenter longitudinal studies before clinical application.

## 1 Introduction

Diabetic retinopathy (DR) is a common microvascular complication of diabetes and an important cause of preventable visual impairment among working-age adults. A global meta-analysis estimated that DR affected approximately 22.3% of individuals with diabetes, corresponding to 103.1 million adults worldwide in 2020, with the number projected to reach 160.5 million by 2045 (1). The International Diabetes Federation estimated that approximately 537 million adults were living with diabetes in 2021, a number projected to reach 783 million by 2045, underscoring the growing population at risk of DR (2).

Chronic hyperglycemia and elevated blood pressure are established determinants of DR onset and progression (3). These systemic risk factors, however, do not directly characterize the retinal microvascular damage present in an individual eye. Clinical DR grading relies on visible retinal lesions (4), whereas capillary perfusion may be impaired before a change in the clinical severity scale becomes apparent. Imaging markers that reflect this microvascular damage may therefore provide additional information when estimating the risk of subsequent worsening.

Optical coherence tomography angiography (OCTA) enables noninvasive, depth-resolved assessment of the retinal circulation. In diabetic eyes without clinically detectable retinopathy, de Carlo et al. (5) identified foveal avascular zone remodeling and capillary nonperfusion that were not evident on routine clinical examination. Subsequent longitudinal studies confirmed these findings. Sun et al. (6) reported that a larger foveal avascular zone, lower vessel density, and lower fractal dimension in the deep capillary plexus were independently associated with DR progression over a median follow-up of more than 2 years. Baseline OCT and OCTA measurements have also been examined in relation to DR development and worsening in other cohorts (7). More recently, a multiethnic prospective study showed that selected retinal OCTA features were associated with 2-year DR progression and improved discrimination when added to clinical variables (8). The longitudinal evidence to date has nevertheless been derived largely from macular OCTA measurements. In diabetic patients without clinical retinopathy, longitudinal OCTA has shown a faster decline of parafoveal superficial vessel density in eyes that subsequently develop retinopathy (9). Combined OCT and OCTA quantification has further identified retinal neurovascular injury in diabetic patients without clinically detectable retinopathy (10). Quantitative OCTA parameters are also altered in eyes with only peripheral diabetic retinopathy, supporting assessment beyond the macular region (11).

The peripapillary circulation may provide information that is not captured by macular imaging alone. Peripapillary microvascular abnormalities have been observed in diabetic eyes, including eyes without clinically apparent DR, and have been related to retinal nerve fiber layer changes (12). In a 3-year prospective study, lower baseline vessel density in the radial peripapillary plexus was associated with subsequent DR progression (13). However, it is not well established whether sectoral peripapillary vessel density provides complementary information when evaluated together with macular deep capillary plexus measurements.

Systemic inflammatory status may represent another component of DR risk. Two large longitudinal studies found that a higher neutrophil-to-lymphocyte ratio was associated with incident DR in patients with type 2 diabetes who did not have retinopathy at baseline (14, 15). These studies addressed the onset of DR rather than the worsening of established retinopathy and did not examine whether routinely available blood-cell measurements added information to OCTA-derived microvascular parameters.

In addition to the mean level of glycemia, long-term visit-to-visit variability of glycated hemoglobin has been associated with an increased risk of retinopathy (16). The relative contributions of mean HbA1c and HbA1c variability differ across the spectrum of diabetic microvascular complications, with retinopathy being most closely related to mean glycemia (17). Diabetic kidney disease and retinopathy show only partial concordance in patients with type 2 diabetes, supporting the independent assessment of renal and retinal microvascular complications (18). Genome-wide polygenic risk scores have also shown independent associations with diabetic retinopathy, indicating a polygenic contribution to individual susceptibility (19). Genetic determinants of glycated hemoglobin additionally influence retinopathy risk, and these effects differ across genetic ancestries (20). In addition, increasing evidence suggests that routinely available hematologic markers may reflect systemic inflammatory status. However, whether these markers are independently associated with the progression of established DR and whether they provide complementary information beyond retinal OCTA parameters remain uncertain.

In this cohort study, we examined the associations of baseline macular and peripapillary OCTA metrics and systemic hematologic biomarkers with 12-month DR progression. We also evaluated the discriminative performance of individual markers, a model combining macular and peripapillary OCTA metrics, and a model that additionally incorporated peripheral blood markers. Given the exploratory nature of this single-center cohort and the limited sample size, these analyses are best positioned as biomarker discovery with preliminary risk-prediction evaluation. Although the rationale for integrating OCTA-derived and hematologic markers is promising, OCTA-based biomarkers are not yet ready for routine prognostic use; reproducibility, segmentation artifacts, device-related differences, and the lack of standardization (21) remain important barriers that must be resolved before clinical implementation can be considered.

## 2 Methods

### 2.1 Study population

Adults aged ≥18 years were eligible if they had a confirmed diagnosis of type 2 diabetes, and diabetic retinopathy in at least one eye at baseline. Participants were excluded for active systemic infection or inflammatory disease, autoimmune or hematologic disease, active malignancy, end-stage renal disease requiring dialysis, decompensated liver disease, or recent treatment with systemic corticosteroids, immunosuppressive agents, or chemotherapy. Eyes were excluded for ocular conditions other than diabetic retinopathy that could affect macular or peripapillary microcirculation or interfere with retinopathy grading, including non-diabetic retinal vascular disease, non-diabetic macular disease, uveitis, glaucoma, or optic neuropathy. Eyes with ungradable fundus or OCTA images because of media opacity, insufficient signal quality, severe motion artifacts, or uncorrectable segmentation errors, as well as those with incomplete baseline or 12-month follow-up data, were excluded.

Of 63 patients screened, 8 were excluded before enrollment (3 because baseline laboratory results were unavailable and 5 because of ungradable OCTA images); 55 patients contributing 108 eyes were enrolled. All 55 participants completed the 12-month follow-up visit (100%); no participant was lost to follow-up.

### 2.2 Ophthalmic examinations

At baseline, all participants underwent a comprehensive ophthalmic examination. Best-corrected visual acuity (BCVA) was measured under standardized illumination using a logarithmic visual acuity chart and converted to logarithm of the minimum angle of resolution values (logMAR) for analysis.

Central macular thickness (CMT) was measured using a spectral-domain OCT system (Heidelberg Engineering, Heidelberg, Germany) and was defined as the retinal thickness from the internal limiting membrane to the retinal pigment epithelium within the central 1-mm-diameter area. Each eye was measured three times, and their average was used for analysis.

Standardized OCTA scans were also acquired at baseline. We collected images of the superficial capillary plexus, deep capillary plexus, and radial peripapillary capillary plexus and used with manufacturer-defined segmentation boundaries. All segmentation results were subsequently reviewed on each B-scan by the investigators. When segmentation errors caused by retinal edema or other anatomical distortions were identified, the segmentation boundaries were manually corrected before quantitative analysis. Quantitative OCTA analysis was subsequently performed offline using a custom Python-based image-processing program, following the general framework of previously reported Python-based regional quantitative analysis of Spectralis OCTA images (22). All images were cropped to the effective scan area and resampled to a standardized spatial resolution of 300 pixels/mm. The images were converted to grayscale and enhanced using contrast-limited adaptive histogram equalization (CLAHE). Vessel segmentation was performed using the Phansalkar local adaptive thresholding algorithm (23). Macular vessel density was measured within the 1–3-mm parafoveal annulus, whereas radial peripapillary capillary vessel density was measured within the 1.5–2.5 mm peripapillary annulus and its superior, inferior, nasal, and temporal sectors. Vessel density was calculated as the proportion of flow-positive pixels within the corresponding region of interest and expressed as a percentage. OCTA and structural OCT images were acquired with a Heidelberg Engineering Spectralis HRA2 platform (Family Acquisition Module 6.9a; Eye Explorer 1.10.2.0; serial number H2E-12052-016-007). Macular and peripapillary scans each covered a 3.0 × 3.0-mm area (10 × 10 degrees). The signal strength index was recorded for every scan; all included images met the quality threshold of the study protocol. For quantification of the deep capillary plexus, projection-artifact removal was applied using the device software. In the custom pipeline, vessel density was defined as the vessel area density (percentage of pixels classified as vessels within the region of interest), and vessel skeleton density was reported separately. Binarization used Phansalkar local adaptive thresholding with k = 0.25, R = 0.5, *p* = 2.0, and q = 10.0; the local window radius was converted from physical scale to pixels at 300 pixels/mm and rounded to an odd integer. Because the installed acquisition software did not provide automated vessel-density measurements for the plexus- and sector-specific parameters analyzed here, agreement with a device built-in software could not be assessed; instead, the reliability of the pipeline was established by intra-operator repeatability、inter-operator agreement and robustness to signal strength.

Standardized dilated ultrawide-field fundus images were acquired at baseline and at 12 months using a scanning laser ophthalmoscope (Optos plc, Dunfermline, United Kingdom). DR severity was graded according to the Early Treatment Diabetic Retinopathy Study Diabetic Retinopathy Severity Scale. Two masked vitreoretinal specialists independently graded the images without access to the participants’ information. The DRSS grading system was applied to ultrawide-field images using a global ETDRS grading approach. This adaptation is supported by published evidence that DRSS grades derived from ultrawide-field imaging agree with standard ETDRS 7-field grading within one step in approximately 92% of eyes and that ultrawide-field imaging additionally captures predominantly peripheral lesions associated with higher rates of progression (24). Inter-grader agreement was excellent: linear-weighted kappa 0.955 for baseline DRSS and 0.987 for 12-month DRSS, and the two graders agreed on the progression classification in 98.1% of eyes (kappa 0.963). Disagreements were resolved by consensus discussion, with adjudication by a senior vitreoretinal specialist when consensus could not be reached; the graders were masked to clinical data, laboratory results, OCTA metrics, and DR progression status.

The primary outcome was DR progression at 12 months. Progression was defined as worsening by at least two DRSS steps relative to baseline, while eyes not meeting this criterion were classified as no DR progression.

### 2.3 Laboratory assessment

At baseline, 5 mL of venous blood was collected on the same day as the ophthalmic examination and analyzed by the institutional clinical laboratory using standardized automated assays. Laboratory measurements included a complete blood count, glycated hemoglobin, liver and renal function indices, and coagulation parameters.

The neutrophil-to-lymphocyte ratio (NLR) was calculated by dividing the absolute neutrophil count by the absolute lymphocyte count. The systemic immune-inflammation index (SII) was calculated as platelet count×neutrophil count/lymphocyte count. The fibrosis-4 index (FIB-4) was calculated as age (years) × aspartate aminotransferase/[platelet count×$\sqrt{\mathrm{alanine}\;\mathrm{aminotransferase}}$ ]. The estimated glomerular filtration rate (eGFR) was calculated using the 2009 CKD-EPI creatinine equation.

### 2.4 Statistical analysis

Statistical analyses were performed using R (version 4.6.1; R Foundation for Statistical Computing, Vienna, Austria) and IBM SPSS Statistics (version 28.0; IBM Corp., Armonk, NY, United States). Continuous variables are presented as mean ± standard deviation or median and interquartile range based on their distributions. Categorical variables are presented as numbers and percentages.

The eye was used as the unit for analysis. Generalized estimating equation models were used to evaluate associations between baseline variables and 12-month diabetic retinopathy progression, with participant identification specified as the clustering variable to account for inter-eye correlation. A multivariable GEE model was used to identify baseline factors independently associated with 12-month DR progression. Receiver operating characteristic curves (ROC) were generated for the individual markers and combined models, and discriminative performance was evaluated using the area under the curve and its 95% confidence interval. All tests were two-sided, and *P* < 0.05 was considered statistically significant. Because the study was exploratory and no formal a-priori sample-size calculation was performed, this is explicitly stated in the Methods and Discussion. For variable selection and validation, LASSO penalized logistic regression (glmnet) with 10-fold cross-validation was performed at the participant level, with both eyes of each participant assigned to the same fold. Internal validation of the final model used participant-level cluster bootstrap (500 resamples) to estimate optimism and optimism-corrected AUCs, together with 10-fold participant-level cross-validation repeated five times. The difference between the AUCs of the OCTA-only and OCTA-plus-lymphocyte models was tested with the DeLong method and with a cluster-bootstrap test that resampled participants to account for inter-eye correlation. Calibration was assessed with a five-group calibration table, a Hosmer-Lemeshow-type statistic, the Brier score, and a bootstrap shrinkage factor. Decision-curve analysis was used to estimate net benefit across clinically plausible threshold probabilities. Sensitivity analyses excluded eyes that received any ocular treatment during follow-up or that required manual segmentation correction, and restricted the model to eyes without diabetic macular edema and to NPDR eyes. Because the four peripapillary sectoral vessel densities were considered jointly in the multivariable analysis, multicollinearity was assessed using pairwise Pearson correlations among the sectoral values and variance inflation factors of the fitted model.

## 3 Results

### 3.1 Study population

A total of 55 patients with type 2 diabetes, contributing 108 eyes, were included. The mean age was 58.69 ± 10.56 years (range 25–78 years). Baseline demographic and clinical characteristics are summarized in Table 1. Both eyes were eligible in 53 patients (96.36%). In the remaining two patients (3.64%), only one eye was included because of vitreous hemorrhage or inadequate OCTA image quality in the excluded eye. All participants completed the baseline ophthalmic, OCTA, and laboratory assessments and the 12-month DRSS evaluation.

**Table 1:** Baseline participant characteristics by 12-month DR progression status.

Variable	Progressors (*n* = 52)	Non-progressors (*n* = 56)
Age (years)	59.17 ± 11.04	58.25 ± 10.18
Diabetes duration (years)	9.97 ± 6.58	10.43 ± 6.34
HbA1c (%)	8.34 ± 2.33	8.21 ± 1.68
BMI (kg/m^2^)	23.40 ± 2.93	23.68 ± 3.23
WBC (×10^9^/L)	6.38 ± 1.51	6.94 ± 1.68
Lymphocyte count (×10^9^/L)	1.78 ± 0.61	2.22 ± 0.57
NLR	2.66 ± 1.57	1.86 ± 0.60
eGFR (mL/min/1.73 m^2^)	81.66 ± 24.94	90.49 ± 21.94
Hypertension, *n* (%)	17 (32.7%)	22 (39.3%)

Data are presented as mean ± standard deviation or number (percentage). DR progression was defined as a worsening of ≥2 steps on the DRSS from baseline to the 12-month follow-up visit.

BMI, body mass index; DR, diabetic retinopathy; DRSS, diabetic retinopathy severity scale; eGFR, estimated glomerular filtration rate; HbA1c, glycated hemoglobin; NLR, neutrophil-to-lymphocyte ratio; WBC, white blood cell count.

At baseline, clinically evident diabetic macular edema was present in 82 of 108 eyes (75.9%), and suspended scattering particles in motion (SSPiM; graded as present or absent on OCT) (25) was present in 17 eyes (15.7%). Signal strength did not differ between progressors and non-progressors (OCT: 34.1 ± 5.6 vs. 34.4 ± 5.3, *P* = 0.80; OCTA macular: 33.4 ± 3.9 vs. 33.1 ± 4.3, *P* = 0.73; OCTA peripapillary: 32.3 ± 3.5 vs. 32.2 ± 4.3, *P* = 0.89).

During the 12-month follow-up, 54 eyes (50.0%) received anti-VEGF injections (1–7 injections; in every treated eye the first injection was administered within the first month), 52 eyes (48.1%) received panretinal photocoagulation, 7 eyes (6.5%) received intravitreal corticosteroid, and 1 eye (0.9%) underwent pars plana vitrectomy; 37 eyes (34.3%) received no ocular treatment. Among the 52 eyes with DR progression, 34 (65.4%) had received anti-VEGF therapy. No eye had received ocular treatment before baseline.

At 12 months, 52 eyes (48.15%) met the predefined criterion for DR progression, whereas 56 eyes (51.85%) did not. Among the 53 patients with both eyes included, bilateral progression occurred in 20 patients, unilateral progression in 12, and no progression in either eye in 21. Neither of the two patients with only one eligible eye showed progression. Overall, 32 patients (58.18%) experienced DR progression in at least one eye.

### 3.2 Associations between baseline factors and 12-month DR progression

The results of the univariable GEE analyses are presented in Table 2. A higher peripheral blood lymphocyte count was associated with lower odds of 12-month DR progression (OR=0.25, 95% CI: 0.10–0.61, *P* < 0.01), whereas a higher neutrophil-to-lymphocyte ratio was associated with higher odds of progression (OR=2.36, 95% CI: 1.25–4.47, *P* = 0.01). Baseline DRSS was also associated with the progression outcome (OR=0.96, 95% CI: 0.92–0.99, *P* = 0.01).

**Table 2:** Univariable analyses of baseline factors associated with 12-month diabetic retinopathy progression.

Characteristics	Unit or Reference	OR (95% CI)	*P*-value
Age	Year	1.01 (0.96–1.06)	0.74
Sex	Female as reference	2.42 (0.91–6.44)	0.08
Duration of type 2 diabetes	Year	0.99 (0.92–1.06)	0.76
Hypertension	No as reference	1.33 (0.49–3.61)	0.57
BMI	kg/m^2^	0.97 (0.83–1.14)	0.71
Blood indicators			
HbA1c	1%	1.04 (0.81–1.32)	0.78
Red blood cell count	1.0 × 10^12^/L	0.83 (0.32–2.11)	0.69
White blood cell count	1.0 × 10^9^/L	0.80 (0.61–1.06)	0.11
Lymphocyte count	1.0 × 10^9^/L	0.25 (0.10–0.61)	<0.01**
NLR	-	2.36 (1.25–4.47)	0.01*
FIB-4	-	1.05 (0.92–1.21)	0.48
eGFR	mL/min/1.73 m^2^	0.98 (0.96–1.01)	0.15
Ocular parameters			
BCVA	0.1 logMAR	0.91 (0.68–1.23)	0.54
Baseline DRSS	Per 1-step	0.96 (0.92–0.99)	0.01*
CMT	*Μ*m	1.00 (1.00–1.00)	0.10
SSPiM	No as reference	1.05 (0.40–2.76)	0.92
Macular OCT parameters			
FAZ area	mm^2^	1.17 (0.37–3.71)	0.79
FAZ circularity Index	-	1.81 (0.28–11.66)	0.53
Parafoveal VD			
Superficial capillary plexus VD	%	1.00 (0.95–1.06)	0.88
Deep capillary plexus VD	%	0.86 (0.80–0.94)	<0.01**
Peripapillary RPC VD			
Temporal VD	%	0.86 (0.81–0.92)	<0.01**
Nasal VD	%	0.94 (0.91–0.98)	0.01*
Superior VD	%	0.95 (0.91–0.99)	0.01*
Inferior VD	%	0.95 (0.91–0.99)	0.02*

Univariable generalized estimating equation (GEE) models were fitted separately for each baseline factor, with 12-month DR progression as the binary outcome.

Macular VD was measured within the 1–3-mm parafoveal annulus, with the Superficial capillary plexus defined from the ILM to the outer IPL boundary and the Deep capillary plexus from the outer IPL to the outer OPL boundary. Peripapillary RPC VD was measured within the 1.5–2.5-mm annulus and its four sectors.

BMI, body mass index; HbA1c, glycated hemoglobin; NLR, neutrophil-to-lymphocyte ratio; SII, systemic immune-inflammation index; FIB-4, fibrosis-4 index; eGFR, estimated glomerular filtration rate; BCVA, best-corrected visual acuity; DRSS, diabetic retinopathy severity scale; CMT, central macular thickness; SSPiM, suspended scattering particles in motion; FAZ, foveal avascular zone; VD, vessel density; RPC, radial peripapillary capillaries; ILM, internal limiting membrane; IPL, inner plexiform layer; OPL, outer plexiform layer; OR, odds ratio; CI, confidence interval.

* *P* < 0.05.

** *P* < 0.01.

Among the OCTA parameters, a higher deep vessel density within the 1–3-mm parafoveal annulus was associated with lower odds of DR progression (OR=0.86, 95% CI: 0.80–0.94, *P* < 0.01). Temporal, nasal, superior, and inferior peripapillary vessel densities were also associated with progression, with ORs of 0.86 (95% CI: 0.81–0.92, *P* < 0.01), 0.94 (95% CI: 0.91–0.98, *P* = 0.01), 0.95 (95% CI: 0.91–0.99, *P* = 0.01), and 0.95 (95% CI: 0.91–0.99, *P* = 0.02), respectively. No statistically significant associations were observed for the remaining candidate baseline variables.

### 3.3 Factors independently associated with 12-month DR progression

Baseline DRSS, neutrophil-to-lymphocyte ratio, peripheral blood lymphocyte count, deep vessel density within the 1–3-mm parafoveal annulus, and the superior, inferior, nasal, and temporal peripapillary vessel densities were entered simultaneously into the multivariable GEE model (Table 3).

**Table 3:** Multivariable analysis of baseline parameters associated with 12-month diabetic retinopathy progression.

Variables	*β* coefficient	Standard error	Wald *χ*2	Adjusted OR (95% CI)	*P*-value
Baseline DRSS	−0.04	0.02	2.99	0.96 (0.92, 1.01)	0.08
NLR	0.12	0.41	0.09	1.13 (0.51, 2.52)	0.76
Lymphocyte count	−1.16	0.52	5.00	0.31 (0.11, 0.87)	0.03*
Deep VD within 1–3-mm parafoveal annulus	−0.15	0.05	7.56	0.86 (0.78, 0.96)	0.01*
Superior peripapillary VD	−0.01	0.03	0.06	0.99 (0.94, 1.05)	0.81
Inferior peripapillary VD	−0.01	0.03	0.13	0.99 (0.93, 1.05)	0.72
Nasal peripapillary VD	−0.01	0.03	0.19	0.99 (0.93, 1.05)	0.66
Temporal peripapillary VD	−0.13	0.04	11.21	0.88 (0.81, 0.95)	<0.01**

A multivariable generalized estimating equation (GEE) model with a binomial distribution and logit link function was used, with patient identification specified as the clustering variable to account for inter-eye correlation. DR progression was defined as a worsening of ≥2 steps on the DRSS from baseline to the 12-month follow-up.

DRSS, diabetic retinopathy severity scale; NLR, neutrophil-to-lymphocyte ratio; VD, vessel density; OR, odds ratio; CI, confidence interval.

* *P* < 0.05.

** *P* < 0.01.

After mutual adjustment, peripheral blood lymphocyte count, deep parafoveal vessel density, and temporal peripapillary vessel density remained significantly associated with 12-month DR progression. For each increase of 1.0 × 10^9^/L in lymphocyte count, the adjusted OR for progression was 0.31 (95% CI: 0.11–0.87, *P* = 0.03). Each 1-percentage-point increase in deep parafoveal vessel density was associated with an adjusted OR of 0.86 (95% CI: 0.78–0.96, *P* = 0.01), while each 1-percentage-point increase in temporal peripapillary vessel density was associated with an adjusted OR of 0.88 (95% CI: 0.81–0.95, *P* < 0.01). However, baseline DRSS and neutrophil-to-lymphocyte ratio were not statistically significant after multivariable adjustment. The three retained predictors remained significant after adjustment for any ocular treatment administered during follow-up and after forced adjustment for age, diabetes duration, HbA1c, and eGFR (lymphocyte count aOR 0.22, deep VD aOR 0.82, temporal pVD aOR 0.86). In the NPDR subgroup (70 eyes), the inverse association of baseline DRSS with progression disappeared (univariable GEE OR 1.01, *P* = 0.62), whereas deep VD and temporal pVD remained independently associated with progression. The four peripapillary sectors showed moderate pairwise correlations (Pearson r = 0.36–0.58), and all variance inflation factors in the multivariable model were below 2 (range 1.13–1.93), indicating that multicollinearity was not a serious concern.

### 3.4 Discriminative performance of individual markers and combined models

Receiver operating characteristic curves for the individual markers and combined models are shown in Figure 1. The AUCs were 0.72 (95% CI: 0.63–0.81) for temporal peripapillary vessel density, 0.70 (95% CI: 0.60–0.80) for deep vessel density within the 1–3-mm parafoveal annulus, and 0.70 (95% CI: 0.60–0.80) for peripheral blood lymphocyte count.

**Figure 1:**
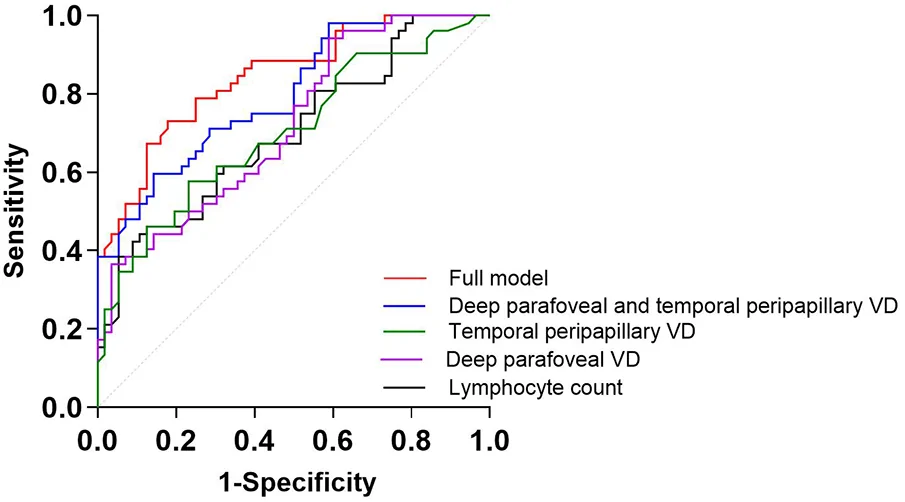
Receiver operating characteristic curves of individual predictors and combined models for discriminating 12-month diabetic retinopathy progression. The AUCs were 0.72 for temporal peripapillary vessel density, 0.70 for deep parafoveal VD within the 1–3-mm parafoveal annulus, and 0.70 for peripheral blood lymphocyte count. The OCTA model, which combined temporal peripapillary vessel density and deep parafoveal vessel density, yielded an AUC of 0.80. The full model, which additionally included lymphocyte count, yielded an AUC of 0.84. Combined-model ROC curves were generated from the predicted probabilities of the corresponding models. DR progression was defined as a worsening of ≥2 steps on the DRSS from baseline to the 12-month follow-up. AUC, area under the curve; DR, diabetic retinopathy; DRSS, diabetic retinopathy severity scale; OCTA, optical coherence tomography angiography; VD, vessel density.

The OCTA model combining temporal peripapillary vessel density and deep parafoveal vessel density yielded an AUC of 0.80 (95% CI: 0.72–0.88). After the addition of lymphocyte count, the AUC of the OCTA-plus-lymphocyte model was 0.84 (95% CI: 0.77–0.92). Internal validation confirmed the incremental value of the lymphocyte count: the difference between the two model AUCs was statistically significant by the DeLong test (*P* = 0.041) and by the participant-level cluster-bootstrap test (mean difference 0.045, 95% CI 0.004–0.117, *P* = 0.026). Calibration of the final model was good (Hosmer-Lemeshow-type statistic 8.14, *P* = 0.42; Brier score 0.162; bootstrap shrinkage factor 0.92).

At the Youden-optimal probability threshold, the OCTA-plus-lymphocyte model correctly classified 73.1% of progressors (sensitivity 0.731, 95% CI 0.59–0.84) and 82.1% of non-progressors (specificity 0.821, 95% CI 0.70–0.91), yielding a positive predictive value of 0.792 (95% CI 0.66–0.89) and a negative predictive value of 0.767 (95% CI 0.65–0.86). Decision-curve analysis further indicated that the combined model provided the highest net benefit across most clinically plausible threshold probabilities (e.g., net benefit 0.28 at a threshold probability of 0.40, compared with 0.22 for the OCTA-only model and 0.14 for treating all patients), supporting its potential clinical utility at the exploratory level.

## 4 Discussion

In this 12-month longitudinal study, lower baseline temporal peripapillary vessel density, lower deep vessel density within the 1–3-mm parafoveal annulus, and lower peripheral blood lymphocyte count were independently associated with DR progression. The AUCs of the individual markers ranged from 0.70 to 0.72, increasing to 0.80 when the two OCTA parameters were combined and to 0.84 after lymphocyte count was added. These findings suggest that regional OCTA measurements and a routinely available systemic marker may provide complementary information on short-term DR progression.

Among participants with both eyes included, 20 showed bilateral progression, 12 unilateral progression, and 21 no progression in either eye. This pattern suggests that DR progression may reflect both systemic factors shared by fellow eyes and eye-specific microvascular differences, although no causal or biological interaction can be inferred from these observations. OCTA-based metrics have revealed subtle inter-eye microvascular asymmetries even in diabetic patients with the same stage of retinopathy in both eyes (26).

### 4.1 Temporal peripapillary vessel density

Temporal peripapillary vessel density showed the strongest association among the OCTA parameters retained in the final model. Subsequent prospective cohorts have also reported associations between lower baseline peripapillary vascular metrics and the incidence or progression of DR, although the populations, OCTA devices, segmentation strategies, and regions of interest differed across studies (13, 27).

The radial peripapillary capillary plexus is closely related to the retinal nerve fiber layer (28, 29). In healthy eyes, its density follows the distribution of nerve fiber bundles and is strongly correlated with retinal nerve fiber layer thickness. In diabetes, reductions in peripapillary vessel density and alterations in capillary morphology have been observed even before clinically evident DR and have been correlated with neural structural changes (12, 30). These observations provide a plausible context for the present temporal-sector finding. Nevertheless, retinal nerve fiber layer thickness, ganglion cell measurements, and functional indices were not evaluated in this study. It therefore remains uncertain whether lower temporal peripapillary vessel density reflects primary microvascular compromise, reduced metabolic demand secondary to neural tissue loss, or a combination of both processes. The ratio of ganglion cell-inner plexiform layer thickness to superficial-vessel density increases with diabetic retinopathy progression (31). OCT-based diabetic retinal neurodegeneration, quantified by macular ganglion cell-inner plexiform layer thickness, independently predicted the development and progression of diabetic retinopathy in a prospective cohort (32). Retinal neurodegeneration progresses steadily over time and differs across risk phenotypes of diabetic retinal disease (33).

### 4.2 Deep parafoveal vessel density

Deep vessel density within the 1–3-mm parafoveal annulus also remained associated with DR progression. This result is consistent with previous longitudinal evidence supporting the prognostic relevance of deep capillary plexus metrics. Sun et al. (6) reported that lower deep capillary plexus vessel density, lower fractal dimension, and a larger foveal avascular zone were independently associated with subsequent DR progression. Other longitudinal cohorts (7) have likewise identified baseline OCT or OCTA features associated with the development or worsening of retinopathy, and a recent multiethnic study (8) found that selected OCTA parameters were associated with 2-year DR progression. In eyes with referable nonproliferative diabetic retinopathy, baseline deep-capillary nonperfusion metrics on OCTA predicted clinically significant complications at one year (34). Deep-learning-based quantification of parafoveal diabetic macular ischemia on OCTA has likewise predicted diabetic retinal disease progression and visual-acuity deterioration (35). Elevated macular choriocapillaris flow deficits on OCTA independently predicted diabetic retinopathy progression and diabetic macular edema development over three years (36). Longitudinal OCTA monitoring has demonstrated progressive retinal vessel closure that tracks stepwise ETDRS severity worsening in nonproliferative diabetic retinopathy (37).

Because cystoid macular edema can distort automated segmentation (38), 82 of 108 eyes (75.9%) required manual correction of segmentation boundaries before vessel-density computation; these eyes were identical to those with clinically evident DME. In a sensitivity analysis restricted to the 26 eyes that did not require manual correction (15 progression events), all three predictors showed directionally consistent associations (lymphocyte count aOR 0.07, deep VD aOR 0.93, temporal pVD aOR 0.83), although none reached statistical significance; this is best explained by the markedly reduced sample size and power (1.9 events per variable; 37 of 52 progressions excluded) rather than instability of the associations. Baseline DME itself was not associated with systematically lower deep-VD values (38.72 ± 6.08 vs. 38.18 ± 5.52 in non-DME eyes, *P* = 0.61), and SSPiM-positive eyes had lower deep VD than SSPiM-negative eyes (35.88 ± 5.06 vs. 39.10 ± 5.96, *P* = 0.019).

The simultaneous retention of temporal peripapillary and deep parafoveal vessel density in the final model suggests that these two parameters capture distinct aspects of retinal microvascular impairment rather than redundant information. The former represents the superficial microcirculation associated with the peripapillary nerve fiber layer, whereas the latter represents the deep parafoveal capillary network. Their combination may therefore capture spatially and anatomically distinct aspects of retinal microvascular impairment. This should be described as statistical complementarity, rather than biological synergy, because no interaction term or mechanistic experiment was performed.

### 4.3 Peripheral blood lymphocyte count

A higher peripheral blood lymphocyte count was associated with lower odds of DR progression and was the only participant-level variable retained in the final model. Most previous longitudinal research has focused on leukocyte-derived ratios rather than the absolute lymphocyte count. Cohort studies in Chinese and Scottish populations have reported that a higher neutrophil-to-lymphocyte ratio was associated with incident DR, although these studies predominantly enrolled patients without retinopathy at baseline and evaluated the development rather than the worsening of established DR (15). A recent systematic review also found associations between several leukocyte-derived indices and DR, but noted substantial methodological and clinical heterogeneity across the included studies (39). Adaptive immune mechanisms, including lymphocyte-mediated responses, have been implicated in the pathogenesis of diabetic retinopathy (40). In a recent longitudinal cohort study, a higher systemic inflammation response index was independently associated with the 2-year development of diabetic retinopathy (41).

The present finding should therefore be regarded as exploratory. An absolute lymphocyte count is readily available in routine clinical practice, but it is nonspecific and may be influenced by age, nutritional status, acute or chronic infection, physiological stress, medication exposure, and other systemic disorders.

NLR was associated with progression in the univariable analysis but was no longer significant after multivariable adjustment, and SII did not meet the conventional significance threshold. These composite indices share the lymphocyte count in their denominator and may also be correlated with other components of the complete blood count. Existing studies of NLR, SII, and related indices have reported heterogeneous results, and most have been cross-sectional or have evaluated incident DR rather than progression of established disease (39). Consequently, the present findings do not exclude a role for systemic inflammation in DR; they indicate only that these composite indices did not contribute independent information within the fitted model. Chronic low-grade inflammation is increasingly recognized as a shared link between neural and vascular impairment in the diabetic retina (42). Cross-sectional studies have associated higher systemic immune-inflammation and systemic inflammatory response indices with the presence and severity of diabetic retinopathy (43).

### 4.4 Discriminative performance and clinical implications

The individual markers showed only modest discrimination, whereas the model combining the two OCTA measurements achieved an AUC of 0.80. The addition of lymphocyte count increased the AUC to 0.84. This pattern is compatible with the premise that measurements obtained from different retinal regions and from the systemic circulation may capture partially distinct information. Previous longitudinal studies have similarly reported improvements in discrimination after adding OCTA parameters to conventional clinical variables.

The present model is viewed as an exploratory combination of candidate markers rather than a clinically validated prognostic tool. Its principal contribution is the identification of a compact group of baseline variables that warrant evaluation in larger longitudinal cohorts.

### 4.5 Strengths and limitations

This study has several strengths, including its longitudinal design, the concurrent assessment of macular and peripapillary OCTA parameters and routinely available hematologic markers, and the use of generalized estimating equations to account for within-participant inter-eye correlation. Several limitations should also be acknowledged. First, this was a single-center study with a relatively small sample size, and participant-level predictors were derived from only 55 individuals. Second, OCTA and laboratory measurements were obtained only at baseline, precluding assessment of longitudinal changes. Third, the follow-up period was limited to 12 months. Finally, the combined model was developed and evaluated in the same dataset. Internal validation (participant-level cluster bootstrap and cross-validation) indicated only minimal overfitting, but no external validation was performed, and calibration was assessed only within the development cohort. The observed discriminative performance should therefore be considered apparent and exploratory, and validation in larger, multicenter prospective cohorts is required. Ocular treatment was administered during follow-up in a majority of eyes, and although the associations remained robust to adjustment for treatment and in untreated eyes, residual confounding by treatment indication cannot be excluded. In eyes with severe nonproliferative diabetic retinopathy, prophylactic intravitreal anti-VEGF therapy can improve diabetic retinopathy severity and reduce progression to vision-threatening complications (44). Because the acquisition software did not provide built-in quantitative vessel-density measurements, agreement between the custom pipeline and a device built-in software could not be assessed; measurement reliability was instead established by intra- and inter-operator reproducibility and robustness to image quality. The calibration and internal-validation results reported here should be confirmed in external cohorts. In addition, although signal strength did not differ between outcome groups and adjustment for the region-matched signal strength did not change the results, vessel-density measurements remain sensitive to residual image-quality variation, media opacity, and device-specific segmentation and quantification algorithms.

## 5 Conclusion

Lower baseline temporal peripapillary vessel density, lower deep vessel density within the 1–3-mm parafoveal annulus, and lower peripheral blood lymphocyte count were associated with higher odds of DR progression over 12 months. The combination of the two regional OCTA parameters and lymphocyte count showed the highest apparent discrimination in the current cohort. These findings support further evaluation of the integration of multiregional OCTA measurements and routinely available hematologic markers for future risk-stratification strategies, although external validation is required before clinical application. These findings are exploratory and hypothesis-generating; the combined model showed promising apparent discrimination that requires external validation before any clinical application.

## Data Availability

The raw data supporting the conclusions of this article will be made available by the authors, without undue reservation.
