# Supplementary material for: Associations of multiregional OCTA metrics and systemic hematologic biomarkers With 12-month diabetic retinopathy progression
Source: Front Med (Lausanne). 2026 Oct 2;13:1951011. doi: 10.3389/fmed.2026.1951011 (PMC13671947; doi:10.3389/fmed.2026.1951011)
Supplement: Supplementary file 1 [file Supplementaryfile1.docx]

SUPPLEMENTARY MATERIALS

Associations of Multiregional OCTA Metrics and Systemic Hematologic Biomarkers With 12-Month Diabetic Retinopathy Progression: A Longitudinal Cohort Study

**Contents**

*Supplementary Materials: contents.*

| **Number** | **Content** | **Cited in** |
| --- | --- | --- |
| Figure S1 | LASSO cross-validation (binomial deviance vs -log-lambda) | Methods 2.4 (penalized regression) |
| Figure S2 | LASSO coefficient paths (23 candidate variables) | Methods 2.4 (penalized regression) |
| Figure S3 | Calibration curve of the final model with bootstrap shrinkage correction | Results 3.4 (calibration) |
| Figure S4 | Cross-validated ROC curves (OCTA vs OCTA + lymphocyte) | Results 3.4 (internal validation) |
| Figure S5 | CONSORT-style participant flow diagram | Methods 2.1 / Results 3.1 |
| Table S1 | GEE analyses restricted to NPDR eyes (n = 70) | Results 3.3 (NPDR subgroup) |
| Table S2 | Pearson correlation matrix of peripapillary sectoral VDs | Methods 2.4 / Results 3.3 |
| Table S3 | Multivariable GEE model forced to adjust for age, diabetes duration, HbA1c, and eGFR | Results 3.3 (forced adjustment) |
| Table S4 | One-eye-per-participant sensitivity analysis (100 resamples) | Methods 2.4 / Results 3.4 |
| Table S5 | Ocular treatment during follow-up and treatment-adjusted sensitivity analysis | Results 3.1 / Results 3.3 |
| Table S6 | Progression rates by baseline DR severity | Results 3.1 (progression rate) |
| Table S7 | Intra- and inter-operator ICCs for all parameters | Methods 2.3 (reliability) |

**Supplementary Table S1**

*Supplementary Table S1. Generalized estimating equation (GEE) analyses restricted to NPDR eyes (n = 70 eyes, 41 participants; 48 progressions).*

| **Variable** | **Univariable OR (95% CI)** | **P** | **Multivariable aOR (95% CI)** | **P** |
| --- | --- | --- | --- | --- |
| Lymphocyte count | 0.27 (0.10-0.79) | 0.016 | 0.40 (0.13-1.21) | 0.106 |
| Deep VD (1-3 mm) | 0.87 (0.78-0.96) | 0.008 | 0.87 (0.77-0.98) | 0.022 |
| Temporal pVD | 0.89 (0.80-0.98) | 0.019 | 0.85 (0.77-0.94) | 0.002 |
| Baseline DRSS | 1.01 (0.96-1.06) | 0.621 | 1.03 (0.95-1.11) | 0.520 |
| NLR | 2.32 (1.07-5.02) | 0.032 | 1.38 (0.55-3.51) | 0.492 |

*GEE with binomial distribution and logit link; participant as clustering variable; independence working correlation. The inverse association of baseline DRSS disappears in the NPDR subset (ceiling effect), whereas deep VD and temporal pVD remain independently associated.*

**Supplementary Table S2**

*Supplementary Table S2. Pearson correlation matrix of peripapillary sectoral vessel densities.*

|  | **Temporal** | **Nasal** | **Superior** | **Inferior** |
| --- | --- | --- | --- | --- |
| Temporal | 1.00 | 0.51 | 0.36 | 0.40 |
| Nasal | 0.51 | 1.00 | 0.58 | 0.44 |
| Superior | 0.36 | 0.58 | 1.00 | 0.42 |
| Inferior | 0.40 | 0.44 | 0.42 | 1.00 |

*Correlations are moderate (r = 0.36-0.58); VIFs of the multivariable model are all < 2 (1.13-1.92), indicating no serious collinearity.*

**Supplementary Table S3**

*Supplementary Table S3. Multivariable GEE model forced to adjust for age, diabetes duration, HbA1c, and eGFR in addition to the retained predictors.*

| **Variable** | **Adjusted OR (95% CI)** | **P** |
| --- | --- | --- |
| Age (per year) | 0.90 (0.82-0.98) | 0.017 |
| Diabetes duration (per year) | 1.04 (0.92-1.17) | 0.512 |
| HbA1c (per 1%) | 1.17 (0.80-1.70) | 0.424 |
| eGFR (per mL/min/1.73 m2) | 0.98 (0.95-1.01) | 0.225 |
| Baseline DRSS | 0.93 (0.88-0.97) | 0.002 |
| NLR | 0.77 (0.34-1.72) | 0.517 |
| Lymphocyte count | 0.22 (0.08-0.63) | 0.005 |
| Deep VD (1-3 mm) | 0.82 (0.72-0.92) | 0.001 |
| Temporal pVD | 0.86 (0.80-0.92) | <0.001 |

*All three retained predictors remained significant after forced adjustment.*

**Supplementary Table S4**

*Supplementary Table S4. One-eye-per-participant sensitivity analysis. In each of 100 resamples, one eye was randomly selected per participant and the multivariable GEE model was refitted.*

| **Outcome** | **Median** | **IQR** | **Consistent direction** |
| --- | --- | --- | --- |
| Lymphocyte count (aOR) | 0.312 | 0.198-0.441 | aOR < 1 in 97.0% of resamples |
| Deep VD 1-3 mm (aOR) | 0.861 | 0.828-0.890 | aOR < 1 in 100% of resamples |
| Temporal pVD (aOR) | 0.889 | 0.857-0.921 | aOR < 1 in 99.0% of resamples |
| AUC of OCTA model | 0.795 | 0.769-0.821 | - |
| AUC of OCTA + lymphocyte model | 0.837 | 0.823-0.862 | - |
| AUC increment | 0.042 | 0.025-0.058 | Positive in 99.0% of resamples |

*This analysis removes the inter-eye correlation entirely and confirms the direction and incremental discrimination of the three retained predictors.*

**Supplementary Table S5**

*Supplementary Table S5a. Ocular treatment during the 12-month follow-up (n = 108 eyes). No eye received ocular treatment before baseline.*

| **Treatment** | **Eyes treated, n (%)** | **Details** |
| --- | --- | --- |
| Anti-VEGF injections | 54 (50.0) | 1-7 injections during follow-up; first within month 1 |
| Panretinal photocoagulation (PRP) | 52 (48.1) | During follow-up |
| Intravitreal corticosteroid | 7 (6.5) | During follow-up |
| Pars plana vitrectomy | 1 (0.9) | After anti-VEGF injections |
| Any ocular treatment | 71 (65.7) | 37 eyes (34.3%) received no ocular treatment |

*Supplementary Table S5b. Multivariable GEE model additionally adjusted for any ocular treatment during follow-up (n = 108 eyes).*

| **Variable** | **Adjusted OR (95% CI)** | **P** |
| --- | --- | --- |
| Baseline DRSS | 0.91 (0.86-0.97) | 0.002 |
| NLR | 1.14 (0.43-3.01) | 0.787 |
| Lymphocyte count | 0.14 (0.03-0.75) | 0.022 |
| Deep VD (1-3 mm) | 0.79 (0.69-0.92) | 0.002 |
| Temporal pVD | 0.82 (0.72-0.93) | 0.002 |
| Any ocular treatment | 69.5 (8.9-542.2) | <0.001 |

*In the 37 eyes without ocular treatment (12 progressions), deep VD remained significant (aOR 0.80, 95% CI 0.66-0.98, P = 0.034); the other predictors were directionally consistent.*

**Supplementary Figure S1**

*LASSO cross-validation curve.*

*Supplementary Figure S1. LASSO cross-validation. Binomial deviance versus -log(lambda) from patient-level grouped 10-fold cross-validation (glmnet convention: -log(lambda) increases from left to right, i.e., lambda decreases and model complexity increases). Vertical dashed lines mark lambda.min (0.059; 7 variables selected) and lambda.1se (0.103; 4 variables selected); the upper axis indicates the number of nonzero coefficients.*

**Supplementary Figure S2**

*LASSO coefficient paths.*

*Supplementary Figure S2. Standardized LASSO coefficients of the 23 candidate baseline variables versus -log(lambda). Colored lines highlight the four variables selected at lambda.1se: baseline DRSS, lymphocyte count, deep parafoveal VD, and temporal peripapillary VD.*

**Supplementary Figure S3**

*Calibration curve of the final model.*

*Supplementary Figure S3. Calibration of the final model. Ideal (dashed), apparent (solid) and bootstrap shrinkage-corrected (dotted) calibration curves. Bootstrap shrinkage factor 0.92; Hosmer-Lemeshow-type statistic 8.14 (df = 8, P = 0.42); Brier score 0.162.*

**Supplementary Figure S4**

*Cross-validated ROC curves.*

*Supplementary Figure S4. Receiver operating characteristic curves from out-of-fold predictions of participant-level 10-fold cross-validation (5 repeats): OCTA model (CV-AUC 0.779 +/- 0.004) vs OCTA-plus-lymphocyte model (CV-AUC 0.810 +/- 0.003).*

**Supplementary Figure S5**

*CONSORT-style participant flow diagram.*

*Supplementary Figure S5. CONSORT-style flow diagram. Of 63 patients screened, 8 were excluded (3 without baseline laboratory results, 5 with ungradable OCTA images); 55 patients (108 eyes) were enrolled. All 55 participants completed the 12-month follow-up; no participant was lost to follow-up.*
